# Insulin Sensitivity and Insulin Resistance in Non-Diabetic Middle-Aged Patients with Obstructive Sleep Apnoea Syndrome

**DOI:** 10.2174/1874192401711010159

**Published:** 2017-12-29

**Authors:** K. Archontogeorgis, N. Papanas, E. Nena, A. Tzouvelekis, C. Tsigalou, A. Voulgaris, M. Xanthoudaki, T. Mouemin, M. Froudarakis, P. Steiropoulos

**Affiliations:** 1 MSc Program in Sleep Medicine, Medical School, Democritus University of Thrace, Alexandroupolis, Greece; 2 Second Department of Internal Medicine, Democritus University of Thrace, Alexandroupolis, Greece; 3 Laboratory of Hygiene and Environmental Protection, Medical School, Democritus University of Thrace, Alexandroupolis, Greece; 4 Division of Immunology, Biomedical Sciences Research Center “Alexander Fleming”, Athens, Greece; 5 Laboratory of Biopathology, University General Hospital of Evros, Alexandroupolis, Greece; 6 Department of Pneumonology, Medical School, Democritus University of Thrace, Alexandroupolis , Greece

**Keywords:** HOMA-IR, Intermittent hypoxia, Insulin resistance, Insulin sensitivity, Obstructive sleep apnoea syndrome, Sleep fragmentation, QUICKI

## Abstract

**Background:**

Obstructive sleep apnoea syndrome **(**OSAS) has been linked with abnormal glucose metabolism, insulin resistance (IR) and development of diabetes mellitus.

**Methods:**

Non-diabetic patients (n=69) with OSAS, diagnosed by polysomnography, were prospectively recruited. To evaluate IR among OSAS patients, the Homeostasis Model Assessment of Insulin Resistance (HOMA-IR) and Insulin sensitivity by Quantitative Insulin sensitivity Check Index (QUICKI) were used.

**Results:**

HOMA-IR was positively associated with body-mass index (BMI) (ρ=0.364, p=0.002), time with oxyhaemoglobin saturation <90% (ρ=0.291, p=0.015), arousal index (ρ=0.268, p=0.027), Epworth sleepiness scale (ESS) score (ρ=0.293, p=0.019) and negatively with average oxyhaemoglobin saturation (ρ=-0.398, p=0.001) and minimum oxyhaemoglobin saturation (ρ=-0.327, p=0.006). QUICKI was positively associated with forced vital capacity (r=0.301, p=0.014), average oxyhaemoglobin saturation (r=0.443, p<0.001), minimum oxyhaemoglobin saturation (ρ=0.318, p=0.008), and negatively associated with sleep stage transitions (r=-0.266, p=0.032), oxygen desaturation index (r=-0.404, p=0.005), time with oxyhaemoglobin saturation <90% (r=-0.311, p=0.019), arousal index (r=-0.344, p=0.004) and ESS score (r=-0.299, p=0.016). After adjustment for age and BMI, HOMA-IR was associated with sleep stage transitions, time with oxyhaemoglobin saturation <90%, average oxyhaemoglobin saturation, minimum oxyhaemoglobin saturation and arousal index. QUICKI was associated with oxygen desaturation index, sleep stage transitions, ESS score, minimum oxyhaemoglobin saturation and arousal index.

**Conclusions:**

An independent association between OSAS and IR in patients without pre-existing diabetes mellitus was observed. Recurrent hypoxia and sleep fragmentation in OSAS are associated with IR in these patients.

## 1 INTRODUCTION

Obstructive sleep apnoea syndrome (OSAS) is characterized by repetitive episodes of partial or complete occlusion of the upper airway during sleep. These episodes are associated with recurrent oxyhaemoglobin desaturation, frequent arousals and sleep fragmentation [1]. Its prevalence is estimated at 10-17% for men and 3-9% for women among adults in developed countries [2]. OSAS is associated with increased prevalence of automobile and occupational accidents, impaired quality of life, reduced work productivity, as well as cardiovascular and cerebrovascular morbidity [3-6].

OSAS has been linked with abnormal glucose metabolism, insulin resistance (IR) and development of type 2 diabetes independently of obesity [7]. IR plays an important role in the development of diabetes mellitus and represents a recognized risk factor for cardiovascular disease [8]. The prevalence of prediabetes in OSAS ranges between 20 and 67% and it is higher than in non-apnoeic subjects [9]. Previous data suggest that exposure to intermittent hypoxia and sleep fragmentation leads to alterations in insulin sensitivity and glucose disposal [10]. However, it is still unclear whether OSAS may lead to the development of diabetes. Additionally, there is still controversy on whether continuous positive airway pressure (CPAP) treatment of OSAS exerts beneficial effects on IR and prevention of diabetes mellitus [11, 12].

The aim of the present study was to investigate the effects of OSAS on IR in patients without diabetes.

## 2 PATIENTS AND METHODS

We included consecutive patients referred to the sleep laboratory of our institution during a 6-month period, between May and November 2016, with symptoms suggestive of sleep-disordered breathing. The study was performed in accordance with the Helsinki Declaration of Human Rights, as revised in 2013 [13], and the patients gave their informed consent.

Exclusion criteria were: known diabetes mellitus; chronic renal or hepatic failure; chronic inflammatory diseases; cancer; systemic steroid treatment and hormonal replacement therapy. Diagnosis of diabetes mellitus was based on medical history or fasting plasma glucose ≥126 mg/dl [14].

Data on previous medical history, current medication use, tobacco smoking and alcohol consumption were recorded. Height, weight, body-mass index (BMI) [weight (kg)/height^2^ (m)], neck, waist and hip circumference and waist/hip ratio were measured using standardized techniques.

Sleepiness was evaluated using the Greek version of the Epworth Sleepiness Scale (ESS) [15], a self-administered questionnaire evaluating the possibility of falling asleep in a variety of situations [maximum score: 24; score >10 indicative of excessive daytime sleepiness]. Pulmonary function testing, arterial blood gases and a 12-lead electrocardiographic examination before sleep study were also performed.

### 2.1 Blood Samples and Measurements

All blood samples were collected after at least 8 h of overnight fasting, were immediately centrifuged (10 min at 3000 rpm, 1116 g) and preserved at -80 C° until analysis. Fasting blood glucose, triglycerides, total cholesterol, high and low density lipoprotein cholesterol, creatinine, and CRP were measured by a random-access chemistry analyser (AU640; Olympus; Hamburg, Germany). The quantitative determination of insulin was carried out using an electrochemiluminescence immunoassay (ECLIA) on a Cobas immunoassay analyser. IR was assessed using the Homeostasis Model Assessment of Insulin Resistance (HOMA-IR = fasting insulin (mU/L) × fasting glucose (mg/dL)/405) and the Quantitative Insulin sensitivity Check Index [QUICKI = 1/(log(fasting insulin µU/mL) + log(fasting glucose mg/dL)] [16, 17].

### 2.2 Polysomnography

Overnight polysomnography **(**PSG) (Alice^®^ 4, Philips Respironics, Murrysville, PA, USA), attended by an experienced technician, was performed from 22:00 to 06:00 hours. A standard montage of electroencephalogram, electro-occulogram, electromyogram and electrocardiogram signals was used. Thoracic cage and abdominal motion were recorded using inductive plethysmography. Pulse oximetry was registered, and airflow was detected using combined oronasal thermistors. The polysomnographic recordings were manually scored using standard criteria [18]. Apnoea was defined as a 90% of reduction in airflow for at least 10 sec [18]. Hypopnoea was defined as a 30% reduction in airflow for at least 10 sec in combination with oxyhaemoglobin desaturation of at least 3% or an arousal registered by the electroencephalogram [18]. The apnoea-hypopnoea index (AHI) was calculated as the average number of apnoeas and hypopnoeas per hour of PSG-recorded sleep time [18]. OSAS was defined as AHI ≥5/h accompanied by related symptoms [1]. OSAS was graded as mild (AHI: 5-14.9/h), moderate (AHI: 15-29.9/h) and severe (AHI ≥30/h) [1].

### 2.3 Statistical Analysis

Analysis was performed using Statistical Package for Social Sciences version 17.0 (SPSS Statistics for Windows, Version 17.0, Chicago, IL). Continuous variables were tested for normality of distribution by the Kolmogorov-Smirnov test. Quantitative data with normal distribution were expressed as mean ± standard deviation (SD) and with skewed distribution as median (25-75^th^ percentile). Correlations were analysed with Pearson’s correlation coefficient, while comparisons between means were explored with the Student t-test. In case of skewed distribution, the Spearman correlation was applied. Associations between variables on univariate regression analysis, with a two-tailed p<0.05, were entered into multivariate models to determine the independent correlations with IR parameters.

## 3 RESULTS

A total of 69 patients (60 males and 9 females), aged 49 ± 12.7 years were included. The anthropometric and sleep characteristics of patients are presented in Tables (**1** and **2**) respectively. Laboratory results and measurements of the study population are presented in Table **3**. Among patients included in the study, 26 (37.7%) were tobacco smokers, 13 (18.8%) were receiving statins and 21 (30.4%) were on anti-hypertensive treatment.

**Fig. (1):**
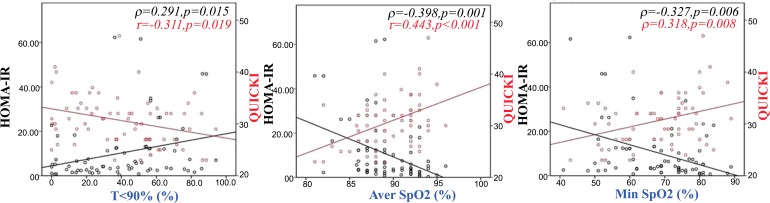
Associations between Quantitative Insulin sensitivity Check Index (QUICKI), Homeostasis Model Assessment of Insulin Resistance (HOMA-IR) and hypoxia parameters (T<90%: Time with oxyhaemoglobin saturation <90%, Aver SpO_2_: Average oxyhaemoglobin saturation, Min SpO_2_: Minimum oxyhaemoglobin saturation) during sleep in obstructive sleep apnoea syndrome patients.

**Fig. (2):**
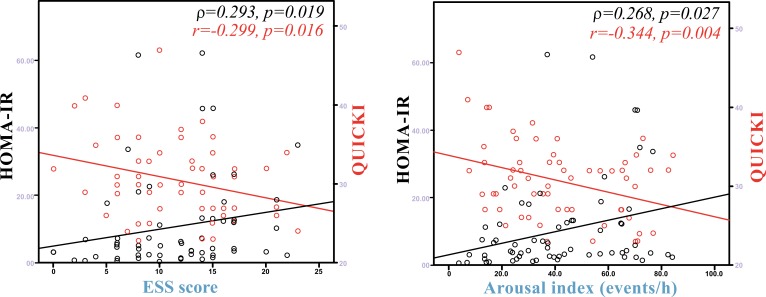
Association between Quantitative Insulin sensitivity Check Index (QUICKI), Homeostasis Model Assessment of Insulin Resistance (HOMA-IR) and Epworth sleepiness scale (ESS) score and arousal index in obstructive sleep apnoea syndrome patients.

HOMA-IR was positively associated with BMI (ρ=0.364, p=0.002), time with oxyhaemoglobin saturation <90% (ρ=0.291, p=0.015) (Fig. **1a**), ESS score (ρ=0.293, p=0.019) (Fig. **2a**), arousal index (ρ=0.268, p=0.027) (Fig. **2b**) and negatively associated with average oxyhaemoglobin saturation (ρ=-0.398, p=0.001) (Fig. **1b**) and minimum oxyhaemoglobin saturation (ρ=-0.327, p=0.006) (Fig. **1c**).

**Fig. (3):**
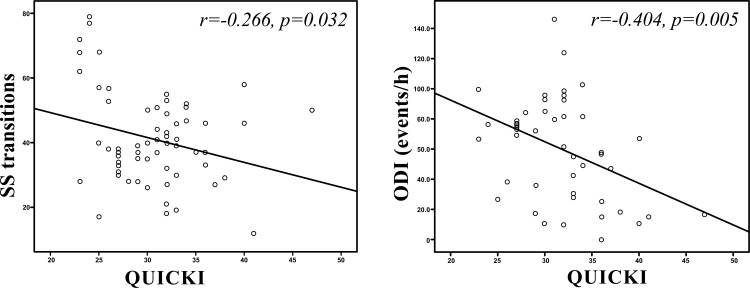
Association between Quantitative Insulin sensitivity Check Index (QUICKI), sleep stage (SS) transitions and oxygen desaturation index (ODI) in obstructive sleep apnoea syndrome (OSAS) patients.

QUICKI was positively associated with forced vital capacity (r=0.301, p=0.014), average oxyhaemoglobin saturation (r=0.443, p<0.001) (Fig. **1b**), minimum oxyhaemoglobin saturation (ρ=0.318, p=0.008) (Fig. **1c**), and negatively associated with sleep stage transitions (r=-0.266, p=0.032) (Fig. **3a**), oxygen desaturation index (r=-0.404, p=0.005) (Fig. **3b**), time with oxyhaemoglobin saturation <90% (r=-0.311, p=0.019) (Fig. **1a**), ESS score (r=-0.299, p=0.016) (Fig. **2a**) and arousal index (r=-0.344, p=0.004) (Fig. **2b**).

There was no association between HOMA-IR, QUICKI and serum lipids, CRP, renal or hepatic function markers. Correlation analysis between HOMA-IR, QUICKI with anthropometric and sleep parameters is presented in Table **4**.

After adjustment for age and BMI, HOMA-IR was associated with sleep stage transitions (β=0.441, p<0.001), time with oxyhaemoglobin saturation <90% (β=0.284, p=0.025), average oxyhaemoglobin saturation (β=-0.400, p=0.001), minimum oxyhaemoglobin saturation (β=-0.374, p=0.002) and arousal index (β=0.256, p=0.041). QUICKI was associated with oxygen desaturation index (β=-0.364, p=0.017), sleep stage transitions (β=-0.259, p=0.041), ESS score (β=-0.255, p=0.047), minimum oxyhaemoglobin saturation (β=0.320, p=0.007) and arousal index (r=-0.304, p=0.012).

Fasting glucose serum levels were positively correlated with proportion of sleep stage 1 in total sleep time (r=0.332, p=0.006) and negatively with minimum oxyhaemoglobin saturation (r=-0.258, p=0.032). There was no association between systolic blood pressure or diastolic blood pressure and any of the sleep parameters.

## 4 DISCUSSION

The major finding of this study is that surrogate markers of IR (evaluated by HOMA-IR and QUICKI) in OSAS patients without diabetes mellitus were associated with indices of hypoxia, sleep fragmentation during sleep, daytime sleepiness and respiratory function.

OSAS *per se* may lead to IR through several intermediary pathways. That process is multifactorial and involves sympathetic overactivity, systemic and adipose tissue inflammation along with hormonal disorders [19, 20]. Sleep deprivation promotes a pro-inflammatory state, with increased levels of interleukin-6, tumour necrosis factor-α and adiponectin, factors also involved in the development of IR [19]. In an animal study, sleep fragmentation reduced insulin sensitivity by increasing macrophage number and infiltration in adipose tissue along with an increase of nicotinamide adenine dinucleotide phosphate oxidase activity [21]. Increased sympathetic tone associated with apnoeic events and intermittent hypoxia has been linked with both IR and metabolic syndrome [22]. Increased catecholamine, cortisol and growth hormone concentrations, described in OSAS, negatively affect insulin activity and glucose metabolism [19].

In the present study, IR was associated with sleep fragmentation, as expressed by increased sleep stage transitions and arousal index. In healthy individuals, it has been shown that sleep fragmentation for two nights decreased insulin sensitivity (p<0.001) and glucose effectiveness (p<0.01), with increased morning cortisol levels and sympathetic activity [23]. In another study involving young healthy adults, selective suppression of slow-wave sleep resulted in decreased insulin sensitivity [24]. Bulcun *et al*. [25] compared disorders of glucose metabolism, evaluated by oral glucose tolerance test between 112 untreated OSAS patients and 19 non-apnoeic control subjects. The rate of disorders of glucose metabolism was higher in OSAS patients compared with controls (50.8 *vs* 15.7%, p=0.005). Arousal index, ESS and BMI were independent significant predictors of IR [25]. The recurrent arousals seen in OSAS patients may have negative impact on glucose metabolism through common pathophysiologic mechanisms activated during sleep deprivation/disruption.

Excessive daytime sleepiness is an established risk factor for the development of both hypertension and diabetes mellitus [26]. In our study, IR was associated with excessive daytime sleepiness, as expressed by the validated ESS questionnaire score. Moreover, our results are consistent with those of previous studies. Barceló *et al*. [27] evaluated IR in 44 OSAS patients matched for age and BMI (22 with and 22 without excessive daytime sleepiness) and 23 healthy controls. IR was assessed by HOMA-IR and excessive daytime sleepiness by the ESS and multiple sleep latency tests. Patients with excessive daytime sleepiness exhibited higher plasma glucose and insulin levels, as well as increased IR, compared with those free from sleepiness and with controls [27]. In the same study, 3 months of CPAP treatment in 35 patients, reduced insulin levels (p=0.03) and HOMA-IR (p=0.007) in subjects with excessive daytime sleepiness, but not in those without excessive daytime sleepiness [27]. In another study that included 25, otherwise healthy, OSAS patients with somnolence and 25 age- and BMI-matched, non-somnolent controls, the former had higher serum glucose (p=0.045), fasting insulin (p=0.012) and HOMA-IR (p=0.027) than the latter [28]. HOMA-IR was positively associated with ESS (p=0.005) and negatively with average oxyhaemoglobin saturation (p=0.031) [28].

Results from the present study indicate an association between hypoxia indices during sleep and IR. The effect of hypoxia on IR has been the subject of interest for previous studies involving OSAS. Ip *et al*. [29] investigated IR in 185 OSAS patients and 85 non-apnoeic subjects. Fasting serum insulin levels and HOMA-IR were higher in OSAS patients compared with healthy controls (p=0.001 and p<0.001, respectively) [29]. Minimum oxygen saturation (p=0.022) and AHI (p=0.044) were independent predictors of HOMA-IR [29]. In another study, enrolling 56 normoglycaemic OSAS patients [30], fasting insulin had shown negative associations with average oxyhaemoglobin saturation (p=0.002) and minimum oxyhaemoglobin saturation (p=0.048), but positive correlations with percentage of time with oxyhaemoglobin saturation <90% (p=0.018) during sleep. HOMA-IR was also correlated with average oxyhaemoglobin saturation levels (r=-0.349, p=0.008) [30]. After 6 months of CPAP therapy, only the patients, who had good adherence to treatment, demonstrated a significant decrease in glycated haemoglobin **(**HbA_1c_) [30]. The underlying mechanism by which intermittent hypoxia induces IR still remains unclear, but recent data suggest that hypoxia-mediated inflammation of the adipose tissue may be an important contributor [31]. Hypoxia is the major determinant of renal function in OSAS patients as well [32, 33].

The QUICKI index is used for the evaluation of insulin sensitivity and is determined by a mathematical transformation, which estimates serum levels of fasting glucose and fasting insulin [17]. In populations other than OSAS, QUICKI has so far demonstrated a better linear correlation with the reference glucose clamp method than other indexes, including HOMA-IR [17, 34, 35]. In OSAS, however, the use of QUICKI is limited mainly to paediatric populations [36].

The present study has several limitations. First, the study population was small and mainly consisted of males referred for sleep disordered breathing. However, this reflects the distribution of consecutive subjects referred to a sleep laboratory related to sleep breathing disorders. Moreover, our data were obtained from middle-aged adults and so we cannot extrapolate results from those findings to older OSAS patients. However, in regression analysis, age was not an influencing factor for the sleep parameters considered. The use of statins, anti-hypertensive drugs and tobacco smoking variably affect insulin resistance and risk of diabetes [37-39]. In the current study, a small number of participants were receiving statins and blood pressure lowering drugs or were tobacco smokers, thus these parameters were not considered in our analysis. Finally, we did not examine the effect of CPAP treatment on glucose metabolism in OSAS patients, a subject that produced conflicting results among studies [40].

In conclusion, our results suggest that OSAS is independently associated with IR in patients without diabetes mellitus. Recurrent hypoxia and sleep fragmentation appear to play an important role in this process. These results should be interpreted in the context of our increasing insight into both the vascular disease risk associated with OSAS [41, 42] and the many comorbidities of the metabolic syndrome [43, 44]. Further studies are needed to evaluate the long-term impact of IR on the incidence of cardiovascular disease in OSAS patients.

## Figures and Tables

**Table 1:** Anthropometric characteristics of obstructive sleep apnoea syndrome (OSAS) patients.

-	**OSAS patients** ***n=69***
**Gender (male/female)**	60/9
**Age (years)**	49 ± 12.7
**BMI (kg/m^2^)**	34.3 (28.7-38)
**Neck circumference (cm)**	43.1 ± 3.7
**Waist circumference (cm)**	122.2 ± 19.2
**Hip circumference (cm)**	117 (110-126)
**WHR**	1.02 ± 0.06
**Smokers (%)**	37.7

BMI: Body Mass Index, WHR: Waist to Hip Ratio

**Table 2:** Sleep characteristics of obstructive sleep apnoea syndrome (OSAS) patients.

-	**OSAS patients** ***n=69***
**Recording time (min)**	380 (352-396)
**TST (min)**	306.1 ± 52.5
**N1 (%)**	15 (8.3-23.4)
**N2 (%)**	68.1 ± 13.1
**N3 (%)**	5.4 (0-11.4)
**REM (%)**	8.6 ± 6.8
**SS transitions**	38.7 ± 13.8
**AHI (events/h)**	65.7 (23.3-83.9)
**ODI (events/h)**	62.9 ± 37.1
**Aver SpO_2_ (%)**	89.9 ± 3.1
**Min SpO_2_ (%)**	69 (56-76)
**T < 90% (%)**	39.2 ± 24.9
**Arousal index**	43.8 ± 20.5
**Sleep efficiency (%)**	86.6 (74.6-91.3)
**ESS score**	10.7 ± 5.6

AHI: Apnoea hypopnoea index, Aver SpO_2_: Average oxyhaemoglobin saturation, ESS: Epworth sleepiness scale, Min SpO_2_: Minimum oxyhaemoglobin saturation, N1: sleep stage 1, N2: sleep stage 2, N3: sleep stage 3, ODI: Oxygen desaturation index, REM: Rapid eye movement, SS transitions: Sleep stage transitions, TST: Total sleep time, T<90%: Time with oxyhaemoglobin saturation <90%.

**Table 3:** Laboratory results and measurements of obstructive sleep apnoea syndrome (OSAS) patients.

-	**OSAS patients** ***n=69***
**FEV_1_ (% predicted)**	92 (72.4-97.6)
**FVC (% predicted)**	83.8 ± 20.7
**FEV_1_/FVC (%)**	82 (79.4-85.1)
**pO_2_ (mmHg)**	78.2 ± 10.5
**pCO_2_ (mmHg)**	40 (38.5-45)
**SBP (mmHg)**	125 (120-130)
**DBP (mmHg)**	80 (75-80)
**Glucose (mg/dL)**	94 (84-120)
**Creatinine (mg/dL)**	0.9 ± 0.16
**Cholesterol (mg/dL)**	197 ± 39
**Triglycerides (mg/dL)**	159 (106-186)
**LDL-C (mg/dL)**	106 (95-129)
**HDL-C (mg/dL)**	46 ± 11
**CRP (mg/dL)**	0.46 (0.22-0.93)
**SGOT (U/L)**	22 (17-31)
**SGPT (U/L)**	32 (18-44)
**Insulin (μU/mL)**	15.6 (8.6-29)
**HOMA-IR**	3.55 (2.23-12.2)
**QUICKI**	0.31 ± 0.06

CRP: C-reactive protein, DBP: Diastolic blood pressure, FEV_1_: Forced expiratory volume in 1^st^ sec, FVC: Forced vital capacity, HDL-C: High density lipoprotein-cholesterol, HOMA-IR: Homeostasis model assessment of insulin resistance, LDL-C: Low density lipoprotein-cholesterol, pCO_2_: Carbon dioxide partial pressure, pO_2_: Oxygen partial pressure, QUICKI: Quantitative insulin sensitivity check index, SBP: Systolic blood pressure.

**Table 4:** Correlation analysis between Homeostasis Model Assessment of Insulin Resistance (HOMA-IR), Quantitative Insulin sensitivity Check Index (QUICKI) and anthropometric and sleep parameters among obstructive sleep apnoea syndrome patients. Correlations are expressed as Spearman rank rho values and as Pearson’s correlation coefficient (*).

-	**HOMA-IR**		**QUICKI**	
	Correlation	p	Correlation	p
**Age**	0.006	0.963	-0.086	0.480*
**BMI**	0.364	**0.002**	-0.347	**0.004**
**Neck circumference**	0.145	0.297	-0.226	0.101*
**Waist circumference**	0.159	0.250	-0.222	0.107*
**Hip circumference**	0.148	0.285	-0.141	0.308*
**WHR**	0.136	0.326	-0.243	0.077
**FEV_1_**	-0.101	0.422	0.115	0.356
**FVC**	-0.158	0.205	0.301	0.014*
**FEV_1_/FVC**	0.006	0.961	0.013	0.916
**pO_2_**	-0.151	0.238	0.233	0.066*
**pCO_2_**	-0.002	0.990	0.158	0.216
**CRP**	0.107	0.387	-0.093	0.452
**TST**	0.218	0.076	-0.085	0.494*
**N1**	-0.051	0.681	0.027	0.827
**N2**	0.206	0.094	-0.112	0.368*
**N3**	-0.226	0.065	0.221	0.072
**REM**	-0.090	0.468	0.093	0.453*
**SS transition**	0.168	0.180	-0.266	0.032*
**AHI**	0.090	0.493	-0.124	0.340
**ODI**	0.397	0.006	-0.404	0.005*
**Aver SpO_2_**	-0.398	**0.001**	0.443	<0.001*
**Min SpO_2_**	-0.327	**0.006**	0.318	**0.008**
**T<90%**	0.291	**0.015**	-0.311	0.019*
**Arousal index**	0.268	**0.027**	-0.344	0.004*
**Sleep efficiency**	0.149	0.228	-0.084	0.500
**ESS score**	0.293	**0.019**	-0.299	0.016*

AHI: Apnoea hypopnoea index, Aver SpO_2_: Average oxyhaemoglobin saturation, BMI: Body mass index, CRP: C-Reactive protein, ESS: Epworth sleepiness scale, FEV_1_: Forced expiratory volume in 1^st^ sec, FVC: Forced vital capacity, Min SpO_2_: Minimum oxyhaemoglobin saturation, N1: sleep stage 1, N2: sleep stage 2, N3: sleep stage 3, ODI: Oxygen desaturation index, pO_2_: Oxygen partial pressure, pCO_2_: Carbon dioxide partial pressure, REM: Rapid eye movement, SS transitions: Sleep stage transitions, T<90%: Time with oxyhaemoglobin saturation <90%, TST: Total sleep time, WHR: Waist to hip ratio.
